# Supplementary material for: Voluntary oral fentanyl intake produces dose- and sex-dependent physical dependence in mice without overt affective disturbances
Source: bioRxiv. 2026 Jul 10:2026.07.06.736848. Preprint. [Version 1] doi: 10.64898/2026.07.06.736848 (PMC13370929; doi:10.64898/2026.07.06.736848)
Supplement: Supplement 1 [file NIHPP2026.07.06.736848v1-supplement-1.pdf]

bioRxiv preprint doi: https://doi.org/10.64898/2026.07.06.736848; this version posted July 10, 2026. The copyright holder for this preprint (which was not certified by peer review) is the author/funder, who has granted bioRxiv a license to display the preprint in perpetuity. It is made available under a CC-BY 4.0 International license.

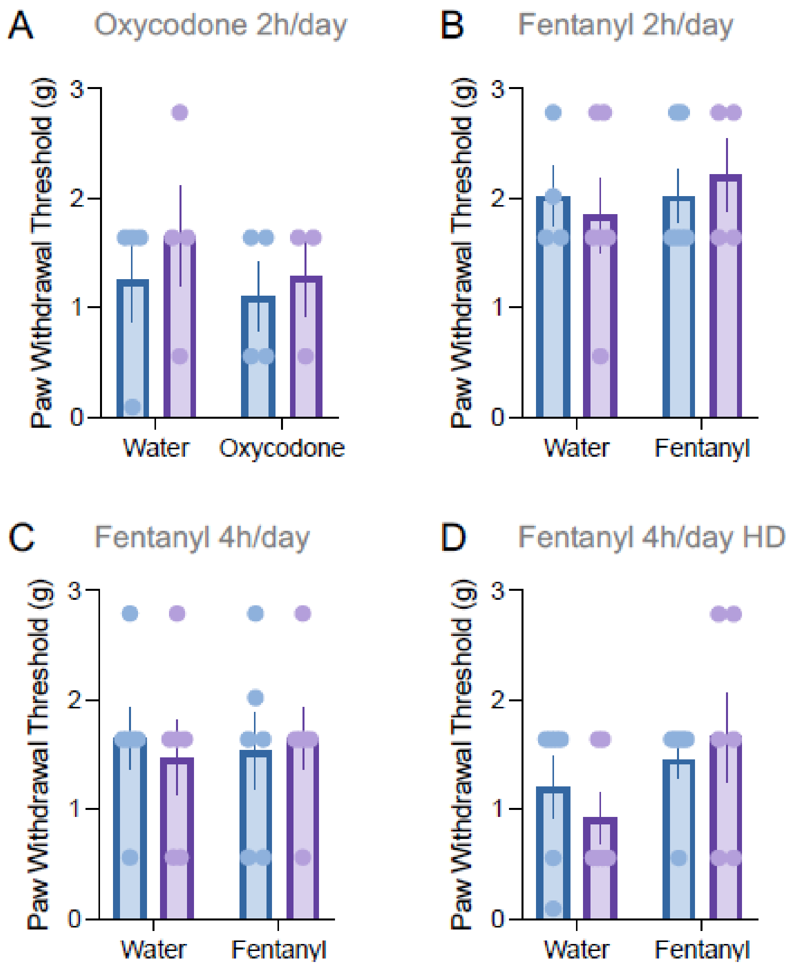

Supplementary Figure 1. Mechanical nociceptive thresholds following oxycodone or fentanyl drinking across cohorts. A. Cohort 1: Oxycodone 2 h/day. Paw withdrawal threshold (g) measured using the von Frey assay in water- and oxycodone-access mice separated by sex (males, blue; females, purple). No significant effects of oxycodone access or sex were observed. **B. Cohort 2: Fentanyl 2 h/day.** Paw withdrawal threshold (g) measured using the von Frey assay in water- and fentanyl-access mice separated by sex. No significant differences in mechanical sensitivity were detected between groups. **C. Cohort 3: Fentanyl 4 h/day.** Paw withdrawal threshold (g) measured using the von Frey assay following 4 h/day fentanyl drinking. No significant effects of fentanyl access or sex were observed. **D. Cohort 4: Fentanyl 4 h/day high-concentration (HD).** Paw withdrawal threshold (g) measured using the von Frey assay in mice exposed to the high-concentration fentanyl drinking paradigm. Mechanical sensitivity did not significantly differ between water- and fentanyl-access groups. Data are presented as mean ± SEM with individual datapoints overlaid. Von Frey testing was used to assess mechanical nociceptive sensitivity following completion of the drinking paradigms.

bioRxiv preprint doi: https://doi.org/10.64898/2026.07.06.736848; this version posted July 10, 2026. The copyright holder for this preprint (which was not certified by peer review) is the author/funder, who has granted bioRxiv a license to display the preprint in perpetuity. It is made available under a CC-BY 4.0 International license.

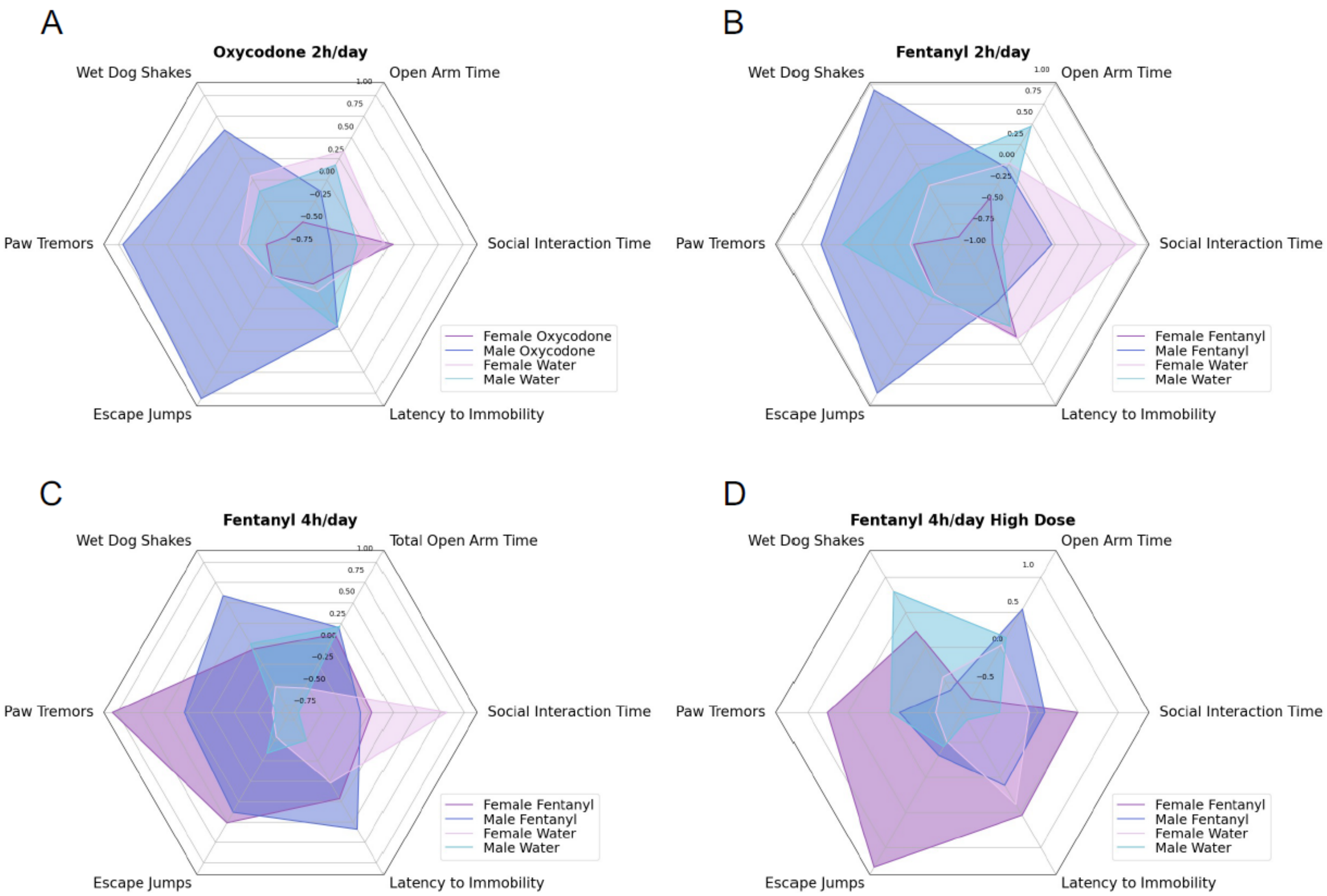

Supplementary Figure 2. Spider plot visualization of behavioral z-scores across oxycodone and fentanyl drinking cohorts. Spider plots depict the mean z-score for each behavioral measure across experimental groups within each cohort following completion of the drinking paradigms. Individual behavioral measures included somatic withdrawal signs (escape jumps, paw tremors, wet-dog shakes), affective-like behaviors (Elevated Zero Maze open arm time, Forced Swim Test latency to immobility, and Social Interaction time), and mechanical sensitivity measured using the von Frey assay. Z-scores were calculated for each behavioral measure and averaged within experimental groups to allow comparison of multidimensional behavioral profiles across cohorts. **A. Cohort 1: Oxycodone 2 h/day.** Oxycodone-access mice exhibited modest increases in withdrawal-associated behavioral z-scores relative to water controls, while affective-like and nociceptive measures remained largely comparable between groups. **B. Cohort 2: Fentanyl 2 h/day.** Fentanyl-access mice demonstrated elevated z-scores for withdrawal-associated behaviors compared to controls, with limited alterations in affective or nociceptive measures. **C. Cohort 3: Fentanyl 4 h/day.** Mice with extended fentanyl access displayed broader increases in withdrawal-related behavioral z-scores relative to water controls, whereas affective-like and mechanical sensitivity measures remained relatively stable. **D. Cohort 4: Fentanyl 4 h/day high-concentration.** High-concentration fentanyl exposure produced the most pronounced behavioral phenotype,

bioRxiv preprint doi: https://doi.org/10.64898/2026.07.06.736848; this version posted July 10, 2026. The copyright holder for this preprint (which was not certified by peer review) is the author/funder, who has granted bioRxiv a license to display the preprint in perpetuity. It is made available under a CC-BY 4.0 International license.

characterized by elevated withdrawal-associated z-scores, particularly in females, with comparatively smaller effects on affective-like behavior and nociceptive sensitivity. Spider plots are intended to provide a multidimensional visualization of group-level behavioral profiles across cohorts and assays rather than individual statistical comparisons.

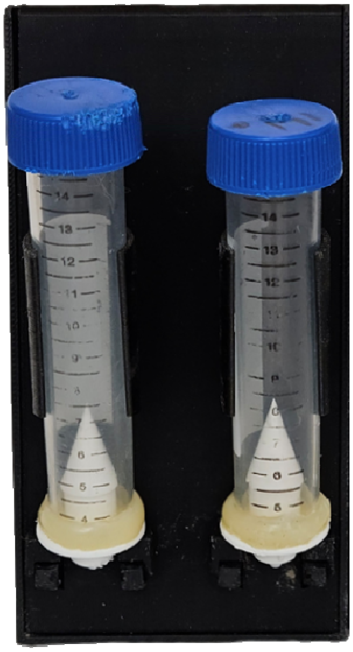

Supplementary Figure 3. Representative image of the 2BC sipper setup.
